# Characterization of Proliferating Lesion‐Resident Cells During All Stages of Atherosclerotic Growth

**DOI:** 10.1161/JAHA.116.003945

**Published:** 2016-08-15

**Authors:** Šárka Lhoták, Gabriel Gyulay, Jean‐Claude Cutz, Ali Al‐Hashimi, Bernardo L. Trigatti, Carl D. Richards, Suleiman A. Igdoura, Gregory R. Steinberg, Jonathan Bramson, Kjetil Ask, Richard C. Austin

**Affiliations:** ^1^ Division of NephrologyDepartment of MedicineMcMaster UniversityHamiltonOntarioCanada; ^2^ Division of EndocrinologyDepartment of MedicineMcMaster UniversityHamiltonOntarioCanada; ^3^ Departments of Pathology and Molecular MedicineMcMaster UniversityHamiltonOntarioCanada; ^4^ Departments of Biochemistry and Biomedical SciencesMcMaster UniversityHamiltonOntarioCanada; ^5^ Department of BiologyMcMaster UniversityHamiltonOntarioCanada; ^6^ Hamilton Centre for Kidney ResearchSt. Joseph's Healthcare HamiltonHamiltonOntarioCanada

**Keywords:** apoptosis, atherosclerosis, cardiovascular disease, lesion, leukocyte, macrophage, proliferation, Atherosclerosis, Vascular Biology, Inflammation, Thrombosis, Animal Models of Human Disease

## Abstract

**Background:**

Monocyte recruitment leads to accumulation of macrophage foam cells and contributes to atherosclerotic lesion growth. Recent studies have reported that lesion‐resident macrophages can proliferate and represent a major cellular component during lesion development. This study was designed to assess whether the rate of macrophage proliferation changes during well‐established stages of lesion growth and to characterize other populations of proliferating cells within these lesions.

**Methods and Results:**

Using murine models of atherosclerosis (*Apoe* ^*−/−*^ and *LDLr* ^*−/−*^ mice) and human coronary artery lesions, in situ proliferation of lesion‐resident cells at different stages of growth was assessed by staining for Ki67 and bromodeoxyuridine (BrdU). In early lesions, close to half of all actively growing macrophages were proliferating in situ. BrdU pulse labeling allowed for accurate identification of in situ proliferating macrophages compared to those derived from monocyte recruitment. Local macrophage proliferation declined as lesions advanced. Interestingly, intimal inflammatory cell infiltrates containing proliferating T lymphocytes were identified during the active phase of lesion growth and correlated with apoptotic cell death. Inflammatory cell infiltrates were completely resolved in advanced lesions and replaced with the necrotic core.

**Conclusions:**

Our findings indicate that atherosclerotic lesions contain locally proliferating macrophages primarily during early and intermediate stages of lesion growth. Furthermore, T‐lymphocyte‐enriched inflammatory cell infiltrates represent a novel subset of proliferating cells within the atherosclerotic lesion that correlate with apoptosis and precede the necrotic core. These findings have novel implications in understanding the pathogenesis of atherosclerosis and may implicate proliferating T lymphocytes as a contributing factor to lesion progression and stability.

## Introduction

Cardiovascular disease (CVD) and its associated ischemic events result from thrombus formation superimposed on disrupted atherosclerotic lesions, a process known as atherothrombosis.^1^ Despite the diversity of cardiovascular risk factors, including genetics, smoking, diabetes mellitus, hypertension, and obesity, the development and progression of atherosclerotic lesions is remarkably comparable.^2^ Endothelial cell dysfunction as well as the accumulation of cholesterol‐rich lipoproteins and expression of proinflammatory factors in the vessel wall are common features of early stages of atherogenesis that result in recruitment of circulating monocytes into the subendothelial space and their differentiation into macrophages.^3^ The unrestricted uptake and accumulation of lipids in these lesion‐resident macrophages eventually leads to formation of foam cells, which enhance lesion growth through secretion of proinflammatory cytokines, cholesterol retention, and apoptosis.^4^ Although apoptosis is considered a protective event in early atherosclerosis, the capacity for clearance and efferocytosis of resident macrophages decreases as the lesions progress, thus contributing to necrotic core formation in advanced disease.^5^ The occurrence and conditions that cause macrophage apoptotic cell death at various stages of lesion development remain poorly defined; however, the intricate and dynamic balance of macrophage death and proliferation is a crucial factor in lesion development and stability.^6^, ^7^, ^8^

In addition to recruitment of circulating monocytes, recent work has demonstrated that local macrophage proliferation is a major contributor to lesion growth, especially as atherosclerosis progresses.^6^, ^9^ However, the relationship between the stage of lesion growth and local macrophage proliferation has not been directly defined.

The concept of local macrophage self‐renewal thus provides a new paradigm in understanding the underlying mechanisms that contribute to atherosclerotic lesion growth and stability.^7^, ^9^, ^10^, ^11^ Despite this important new discovery, the correlation between macrophage proliferation and plaque growth, as well as the involvement of other proliferating lesion‐resident cell types, has not been determined.^10^, ^12^

The aim of the present study was to utilize mouse models of atherosclerosis and archived tissue derived from atherosclerotic patients to further characterize and identify in situ proliferating lesion‐resident cells during all stages of lesion growth. Specifically, we sought to measure whether the rate of macrophage proliferation changes over the course of lesion progression. The use of antigen KI‐67 (Ki67) immunostaining showed the presence of proliferating macrophages within all stages of murine atherosclerotic lesion growth as well as in human coronary artery atherosclerosis. Furthermore, bromodeoxyuridine (BrdU) pulse labeling was used to accurately distinguish locally proliferating macrophages from those derived from circulating monocytes.^13^ Characterization of locally, or in situ, proliferating macrophages shows their abundance predominantly in early lesions of *Apoe* ^*−/−*^ mice on a chow diet as well as *LDLr* ^*−/−*^ mice on a high‐fat diet. Close to half of all replicating macrophages were derived from in situ proliferation as opposed to monocyte recruitment.

Furthermore, the local or in situ proliferation of other leukocytes, including T lymphocytes, and their contribution to plaque growth has not been fully characterized, despite the importance of T lymphocytes in atherothrombosis.^14^, ^15^, ^16^, ^17^ Although adventitial inflammatory infiltrates containing T lymphocytes have been previously reported, their occurrence and role in the intima remains poorly defined.^18^ Our findings demonstrate the presence of transient intimal inflammatory cell infiltrates (ICIs) consisting of proliferating CD3‐positive T lymphocytes, which are associated with lesion growth, apoptosis, and a decrease in macrophage proliferation. Our data highlight the diversity and breadth of lesion resident immune cell proliferation during atherogenesis. The balance between leukocyte proliferation and apoptosis is paramount to development of atherosclerotic lesions, and continued delineation of this complex milieu will aid in further understanding the progression of atherothrombosis at the cellular level.

## Methods

### Animals, Diets, and Reagents

*Apoe* ^*−/−*^ male and female mice on chow diet, from 8 to 55 weeks old, were sacrificed by cervical dislocation under isoflurane anesthesia. Mice were maintained on a control chow diet, as opposed to a high‐fat diet, to reduce the potential confounding effect of obesity and insulin resistance to atherosclerotic lesion development and stability.^19^ A minimum of 10 *Apoe* ^*−/−*^ mice were used from each stage of atherosclerosis (as defined by age and lesion characteristics) with ≈3 to 5 sections per animal for each immunohistochemical (IHC) analysis. For BrdU pulse labeling, mice were injected with the BrdU Labeling Reagent Ready‐to‐use (Cat. No. 000103; Invitrogen, Carlsbad, CA), at 1 mL/100 g body weight intraperitoneally, and the heart and small intestine removed at 2 or 24 hours postinjection. *LDLr* ^*−/−*^ males at 14 weeks of age fed a high‐fat diet (42% of calories from fat, 0.2% cholesterol; TD09268; Harlan Teklad, North Easton, MA) for 6 or 8 weeks were also used for IHC analysis. As an accelerated model of diet‐induced lesion formation, 10‐week‐old *Apoe* ^*−/−*^ mice were made hyperhomocysteinemic by supplementing the drinking water with 0.5% (w/v) l‐methionine (Sigma‐Aldrich, St. Louis, MO), as described by us previously.^20^ All procedures were approved by the McMaster University Animal Research Ethics Board. Hearts containing the ascending aorta were dissected, immersion fixed in formalin, and embedded in paraffin. Serial sections (4 μm thick) were cut and collected from the point of the aortic valve, perpendicular to the axis of the aorta, through the aortic root as long as lesions were observed in the microscope in unstained sections.^21^

### Immunohistochemical

Sections (4 μm thick) were deparaffinized and the endogenous peroxidase activity blocked with 0.5% H_2_O_2_ in methanol for 10 minutes. Antigen retrieval was performed where necessary, sections blocked with 5% normal serum, and incubated with the primary antibody for 1 hour, followed by biotinylated secondary antibody (Vector Laboratories, Burlingame, CA) diluted 1:500, and streptavidin‐peroxidase (Vector Laboratories) diluted 1:50. Sections were developed in Nova Red peroxidase substrate (Vector Laboratories) and counterstained with hematoxylin.

### Immunofluorescence

Sections (4 μm thick) were deparaffinized and antigen retrieval performed, where necessary. After blocking with 5% normal serum, sections were incubated with the first primary antibody for 1 hour, rinsed with Tris buffer, incubated with the second primary antibody for 1 hour, and rinsed with Tris buffer. A mix of Alexa Fluor (Life Technologies, Carlsbad, CA) 488 and 594 secondary antibodies, each diluted 1:200, was used to incubate sections for 30 minutes. Coverslips were then mounted onto each slide using Permafluor.

### Antibodies

Antibodies included anti‐BrdU, ab1893 (Abcam, Cambridge, MA), heat‐induced epitope retrieval (HIER; pH 6.0), used at 1:500; anti‐Ki67, ab16667 (Abcam), HIER, 1:100; anti‐Mac‐3, #55322 (BD Pharmingen, San Diego, CA), HIER, 1:1000; anti‐CD3, A 0452 (DakoCytomation, Carpinteria, CA), HIER, 1:100; anti‐CD45R, #550286 (Pharmingen), HIER, 1:50; anti‐mannose receptor (MR), ab64693 (Abcam), HIER, 1:1000; anti‐YM1 (chitinase 3‐like 3), R&D Systems (Minneapolis, MN), HIER, 1:1000; anti‐iNOS (inducible nitric oxide synthase), ab15323, (Abcam), HIER, 1:100; anti‐arginase I (Arg I), BD Transduction, 1:100; anti‐FOXP3, FJK‐16 (eBioscience, San Diego, CA), HIER, 1:10; anti‐MPO (myeloperoxidase), 120‐15484 (Novus, Saint Charles, MO), HIER, 1:10; anti‐SMA (smooth muscle actin), A2547 (Sigma‐Aldrich), 1:200; anti‐CD68, clone KP1 (DAKO), HIER, 1:100; anti‐CD4, 14‐9766 clone 4SM95 (Affymetrix, Santa Clara, CA), HIER, 1:50; and anti‐CD8, 14‐0808 Clone 4SM15 (Affymetrix), HIER, 1:100. Isotype and preimmune negative controls were used during staining optimization and lacked background staining.

### Terminal Deoxynucleotidyl Transferase dUTP Nick End Labeling Assay

The TACS^®^2 TdT kit (4810‐30‐K; Trevigen, Gaithersburg, MD) was used according to the manufacturer's instructions, followed by immunofluorescence staining.

### Human Coronary Arteries

Sections of human coronary arteries were obtained from tissue blocks containing explanted hearts of transplant recipients or cadaver organ donors. All procedures were approved by the McMaster University Research Ethics Board.

### Statistical Analysis

Statistical analysis between 2 groups was performed using a Mann–Whitney *U* test. Error bars represent SEM, unless otherwise noted. Data were considered statistically different if *P*<0.05 and demarcated by using an asterisk.

## Results

### Proliferating Macrophages Are Present at Various Stages of Murine Atherosclerotic Lesion Development and in Human Coronary Artery Atherosclerosis

**Figure 1:**
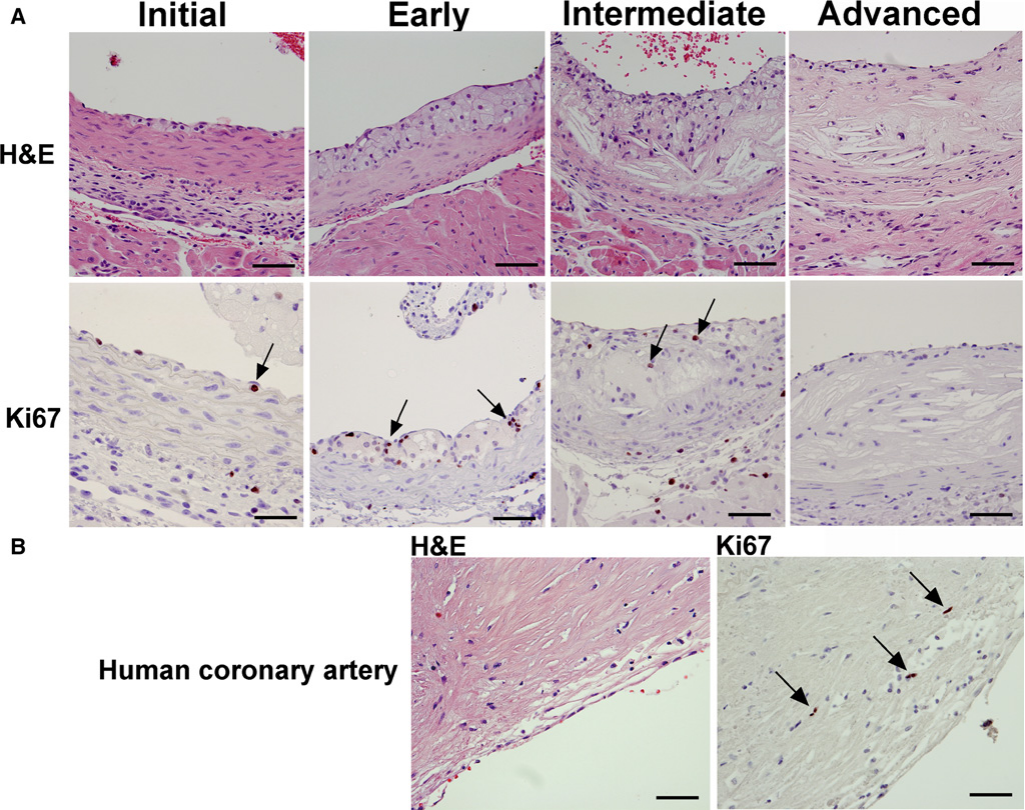
Detection of proliferating cells during murine and human atherosclerotic lesion growth. Ki67 immunohistochemistry and H&E staining in *Apoe* ^*−/−*^ mice on chow diet identified proliferating cells during various stages of lesion progression, ranging from initial macrophage infiltration to advanced lesions (A). In the initial and early stage, macrophages below the endothelium, as well as foam cells in fatty streaks, were Ki67 positive (Initial and Early, arrows). At 24 weeks of age, in lesions of the intermediate type with large necrotic cores, few Ki67‐positive cells were observed (Intermediate, arrows). At 55 weeks of age, lesions were mostly necrotic with very few Ki67‐positive cells (Advanced). Human lesions from coronary arteries stained with Ki67 contained some Ki67‐positive cells in areas rich in macrophages (B, arrows). Bar=50 μm. H&E indicates hematoxylin and eosin.

Paraffin sections of the aortic root from female *Apoe* ^−/−^ mice at different stages of atherosclerosis were stained with hematoxylin and eosin (H&E) to assess lesion growth and gross cellular morphology (Figure 1A, upper panel). Sections were also stained for Ki67, a well‐established marker of active cell division,^22^ to assess the presence of proliferating cells in these different stages of atherosclerosis (Figure 1A, lower panel). At 8 weeks of age, designated the initial stage, the first intimal macrophages were identified in the subendothelial space. At 12 weeks of age, designated the early lesion group, intimal fatty streaks consisting of lipid‐enriched macrophage foam cells were observed. Consistent with these findings, lipid‐enriched macrophage foam cells stained intensely for filipin, a marker of free cholesterol, and Oil Red O (ORO), a marker of neutral lipids (Figure S1). This early stage of lesion growth showed no evidence of apoptotic cell death nor the appearance of a lipid‐enriched necrotic core, consistent with previous findings.^8^ At 24 weeks of age, designated the intermediate stage of lesion growth, the intima consisted of a necrotic lipid core with cellular debris and a cellular/fibrous cap. Stretches of simple fatty streaks or individual intimal macrophages were also observed adjacent to these complex lesions (not shown). Filipin and ORO staining was markedly increased in both the necrotic regions and adjacent intact foam cells (Figure S1). At 55 weeks, designated the advanced stage of lesion growth, the lesions were relatively acellular as defined by lack of nuclei, comprised of only a few intact viable cells.

Ki67 is expressed during all active phases of the cell cycle, but is absent in resting cells, and represents a reliable marker for identifying cells that are in the growth cycle.^22^ A small proportion of intimal cells were Ki67 positive at 8 weeks of age (arrow). Several Ki67‐positive cells were observed in the fatty streaks of the early lesion group (arrows), some of which were foam cells by morphology, a result consistent with filipin and ORO staining of lesions (Figure S1). The intermediate group contained some proliferating cells in the cellular/fibrous cap (arrows); however, very few proliferating intimal cells were observed in the advanced stages of lesion growth. Advanced human atherosclerotic plaques from coronary arteries also contained several Ki67‐positive cells, both near the lumen (Figure 1B, arrows) and the medial layer (not shown).

**Figure 2:**
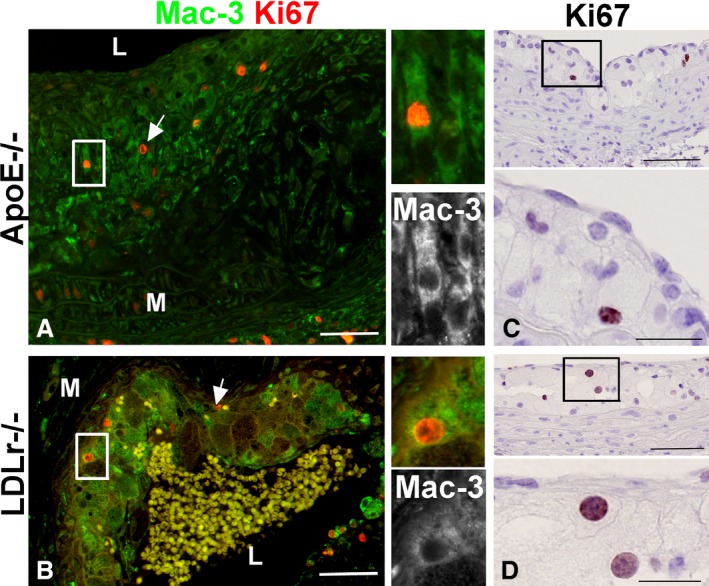
Mac‐3 and Ki67 immunofluorescence of *ApoE* ^*−/−*^ and *LDLR* ^*−/−*^ atherosclerotic lesions detects proliferating macrophages. Double immunofluorescence for the macrophage marker Mac‐3 (green) and Ki67 (red) reveals that some of the proliferating cells in the *Apoe* ^*−/−*^ (A) and *LDLr* ^*−/−*^ (B) lesions were macrophages. By immunohistochemistry, Ki67‐positive cells exhibited foam cell morphology with large nuclei and lipid vesicles visible at high magnification (insets), in fatty streaks from *Apoe* ^*−/−*^ mice (C), and from *LDLr* ^*−/−*^ mice (D). L, lumen; M, media; bar=50 μm, and 20 μm in high‐magnification insets.

**Figure 3:**
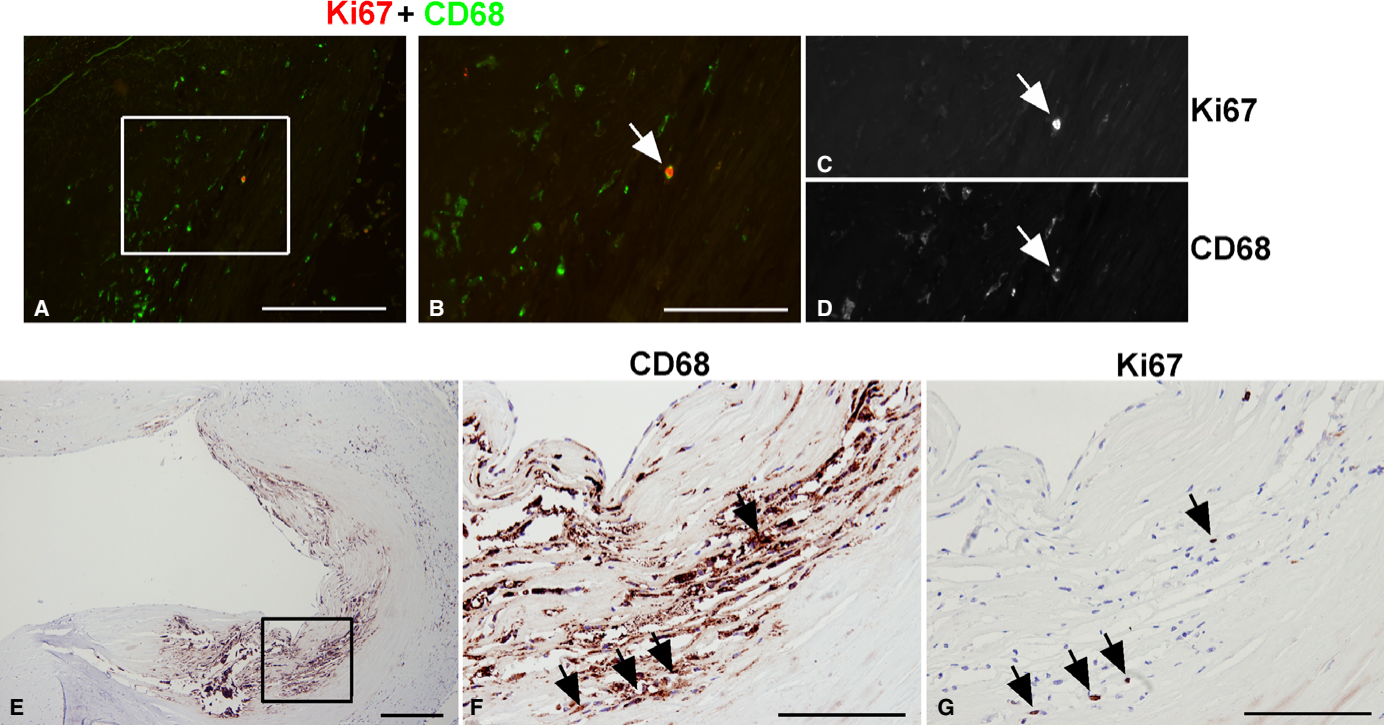
CD68‐ and Ki67‐positive macrophages in human coronary artery atherosclerotic lesions. CD68 staining identified proliferating cells (Ki67 positive) in human coronary arteries as macrophages. Double immunofluorescence of Ki67 and CD68 (A through D), a double‐labeled cell (Ki67, red; CD68, green), was identified in (A) and magnified in (B). The same cell is shown in separate monochrome images (C and D). Lesion areas rich in macrophages were identified by CD68 immunohistochemistry (E) and then analyzed at higher magnification (F). Adjacent sections were stained with Ki67 (G) and showed that Ki67‐positive cells were found in areas rich in macrophages (arrows). Bar=100 μm.

To directly identify murine lesion‐resident proliferating cells as macrophages, double immunofluorescence of aortic sections was used to label Mac‐3‐positive macrophages (green) that expressed Ki67 (red; Figure 2). Ki67‐positive macrophages were detected in the lesions of *Apoe* ^*−/−*^ mice fed a chow diet (Figure 2A) as well as in the lesions of *LDLr* ^*−/−*^ mice fed a high‐fat diet (Figure 2B). The appearance of lipid droplets and the characteristic large, round nuclei further indicates that the Ki67‐positive cells from *Apoe* ^*−/−*^ (Figure 2C) and *LDLr* ^*−/−*^ mice (Figure 2D) were indeed macrophage foam cells. It is important to note that some proliferating cells were not macrophages, as indicated by positive Ki67 staining in the absence of Mac‐3 staining (Figure 2A and 2B, arrows). We also identified lesion‐resident proliferating cells as macrophages in human coronary arteries using Ki67 and CD68 immunostaining (Figure 3, upper panel).^9^, ^23^ Immunofluorescent staining showed that Ki67‐positive cells are also CD68 positive (Figure 3A through 3D), thus identifying locally proliferating macrophages in human atherosclerosis, consistent with findings by Robbins et al.^9^ Furthermore, IHC of macrophage‐rich areas of human lesions illustrated the presence of Ki67‐positive cells (Figure 3E through 3G).

### Lesion‐Resident Macrophages Proliferate In Situ

To further confirm our Ki67 immunostaining results and to assess the origin of the proliferating cells, a BrdU pulse‐labeling experiment was performed in 12‐week‐old (early lesion group) and 24‐week‐old (intermediate group) *Apoe* ^*−/−*^ mice fed a chow diet. BrdU pulse labelling was utilized as a reliable method for identifying in situ proliferation.^24^ BrdU is incorporated into DNA only during the S phase of the cell cycle and has a half‐life of ≈1 hour in the animal.^24^ Thus, all cells that are in the S phase in the 1‐hour window will be labeled, and, once incorporated, BrdU can be detected by IHC in cells that have incorporated it as well as their daughter cells.^13^ Lesion‐resident S‐phase cells, as well as proliferating monocytes derived from the bone marrow and spleen will incorporate BrdU during the pulse. Zhu et al. showed that labeled monocytes can be detected in the bone marrow within 2 hours, and in the blood at 6 hours post‐BrdU pulse injection, and that their number in circulation increases and peaks at 12 to 24 hours.^13^ Therefore, any BrdU‐positive cells in lesions from mice sacrificed at 2 hours postinjection (p.i.) would be cells that are proliferating locally, or in situ, and are not derived from the circulation. Mice sacrificed at 24 hours p.i., however, would contain BrdU‐positive cells that were in S phase both in situ and those that were labeled as monocytes and have since traveled through the circulation, extravasated into the intima, and differentiated. This is consistent with the BrdU‐stained sections of the jejunum at 2 and 24 hours post‐BrdU injection (Figure S2). The intestinal epithelium undergoes turnover as cells are sloughed off from the tips of villi and are replaced by cells that proliferate in the crypts and migrate along the villus.^25^ Indeed, in sections from mice sacrificed at 2 hours p.i., the cells in the crypts were positive, whereas at 24 hours p.i., positive cells were only detected further up along the villus (Figure S2).

**Figure 4:**
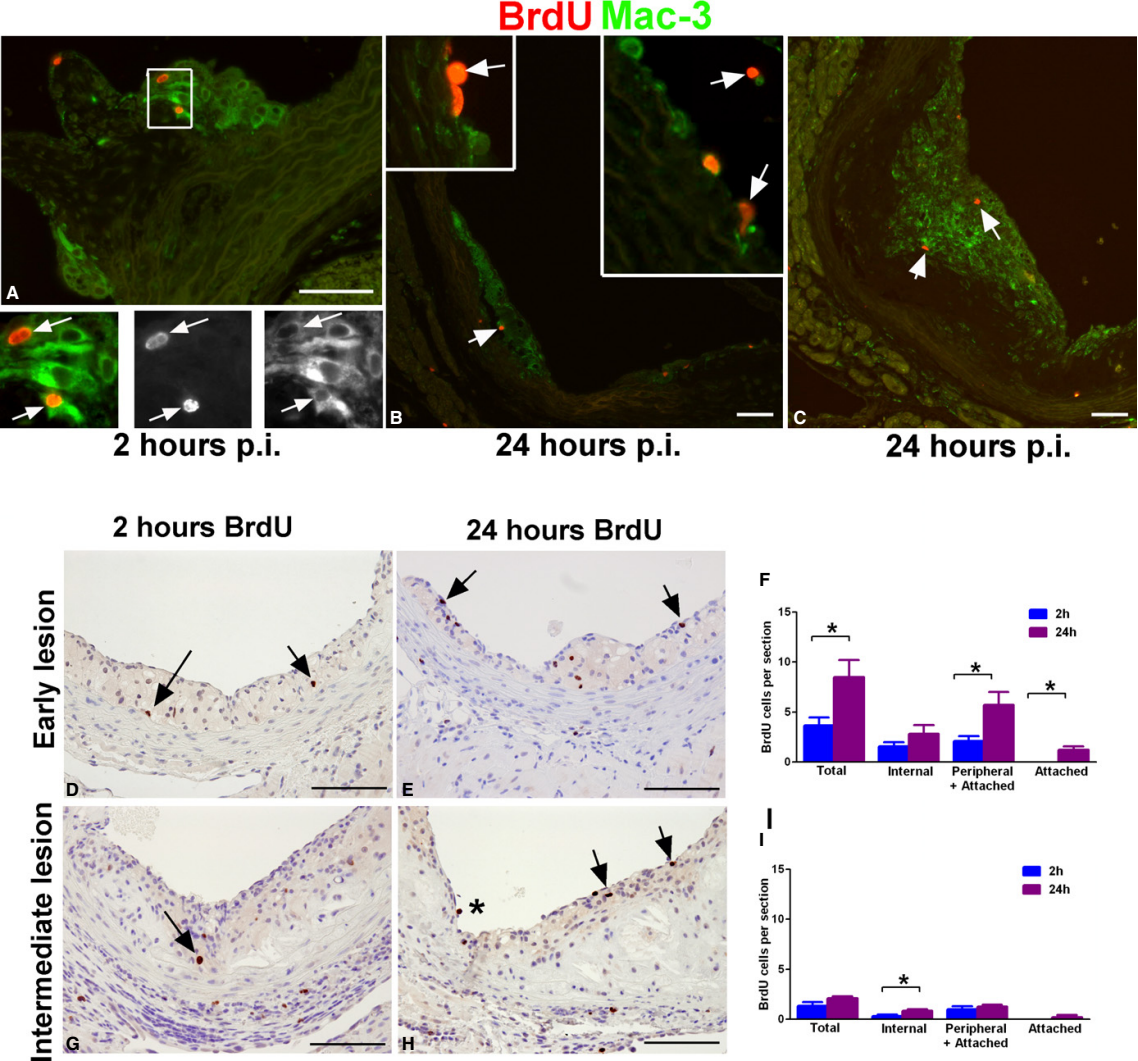
BrdU pulse labeling detects macrophage proliferation in situ and tracks monocyte incorporation into lesions. Mac‐3‐positive macrophages in lesions incorporated BrdU within 2 hours p.i. (A, arrows). At 24 hours p.i., BrdU‐positive monocytes were observed in the lumen, attached to the endothelium or extravasating into the intima (B, arrows in insets), in addition to macrophages within the lesion that had proliferated in situ (B and C, arrows). Aortic root sections from *Apoe* ^*−/−*^ mice on a chow diet at 12  (early, n=11) and 24 weeks (intermediate, n=9) were immunostained for BrdU both 2 and 24 hours after BrdU injection. Early lesion, 2 hours p.i. (n=6) (D) and 24 hours p.i. (n=5) (E). Intermediate lesion 2 hours p.i. (n=4) (G) and 24 hours p.i. (n=5) (H). Arrows indicate BrdU‐positive cells. Positive cells were quantified in 3 sections from each mouse and averaged. BrdU‐positive macrophages were classified based on their location within the lesion as peripheral (close to the endothelium), internal (deeper in the lesion), or attached (in the lumen attached to the endothelium). Quantification of early (F) and intermediate lesions (I). Bar=50 μm. ^*^ *P*<0.05 as measured by Mann–Whitney *U* test. BrdU indicates bromodeoxyuridine; p.i., postinjection.

Aortic sections were immunostained for BrdU and Mac‐3 and visualized by immunofluorescence. In the early lesion group sacrificed at 2 hours p.i. (Figure 4A), some of the Mac‐3‐positive macrophages were observed to express BrdU, a finding consistent with the positive Ki67 immunostaining (Figure 1B), and confirming that intimal aortic macrophages are proliferating locally, or in situ. No BrdU‐positive cells were observed in the lumen of the aorta at this time point. In the early lesion group sacrificed at 24 hours p.i., BrdU‐positive monocytes were identified in the lumen, attached to the endothelium, and extravasating through the endothelial layer (Figure 4B, arrows). BrdU‐positive cells were also observed in intermediate lesions, some located in the basal portion similar to the 2‐hour time point, but mostly located toward the luminal aspect of the lesion (Figure 4C, arrows).

By scoring and comparing BrdU‐positive cells at 2 and 24 hours p.i. in aortic sections, the relative contribution of in situ proliferation and of monocyte recruitment into the lesion was determined (Figure 4D through 4I). BrdU‐positive macrophages were classified based on their location within the lesion as peripheral (close to the endothelium), internal (deeper in the lesion), or attached (in the lumen attached to the endothelium). The early lesion group consisted of extensive fatty streaks without any morphological evidence of apoptotic cell death (Figure 4D and 4E). The mean total number of BrdU‐labeled cells per section was 8.50±1.71 at 24 hours compared to 3.64±0.80 at 2 hours (*P*=0.022), suggesting that ≈57% of the BrdU‐positive cells were derived from the bone marrow (Figure 4F). These data show that 43% of all proliferating cells in early lesions were undergoing proliferation in situ (Figure 4F). No BrdU‐positive cells were observed attached to the endothelium at 2 hours p.i. versus 1.37±0.31 cells per section at 24 hours, indicating a lack of blood‐derived cells at 2 hours (Figure 4F), as expected. We assumed that BrdU‐positive cells found at the lumenal aspect of the neointima (peripheral) were mostly cells derived from circulating monocytes within the 24‐hour window. Indeed, the total number of peripheral and attached cells was 5.70±0.51 at 24 hours versus 2.08±1.31 at 2 hours (*P*=0.027) per section. As expected, the number of internal cells was not statistically different between the 2 groups given that these cells most likely represent solely in situ proliferation (Figure 4F). These data indicate that newly arrived cells (internal and peripheral) contribute to the statistically significant difference of total BrdU‐positive cells between 24 and 2 hours p.i. in early lesions. In the intermediate lesion group (Figure 4G and 4H), areas of necrosis containing cholesterol crystals were observed. The total number of BrdU‐positive cells per section was much less than in the early lesion group despite a larger lesion area (Figure 4I). Upon analyzing intermediate lesions, the total number of BrdU‐positive cells per section was 2.10±0.19 versus 1.31±0.40 (*P*=0.095) in the 24‐ and 2‐hour p.i. groups, respectively (Figure 4I). This suggests a small degree of local proliferation in intermediate lesions as well as a decreased amount of recruited monocytes compared to early lesions. In fact, only 1 BrdU‐positive cell was found attached to the endothelium in all 15 sections examined (Figure 4H, asterisk), compared to the 17 cells found in the early lesion group, indicating that lesion growth both by in situ macrophage proliferation and monocyte recruitment is considerably reduced in the intermediate lesions from *Apoe* ^*−/−*^ mice fed a chow diet.

### Identification and Characterization of Inflammatory Cell Infiltrates in the Intermediate Stage of Lesion Growth

**Figure 5:**
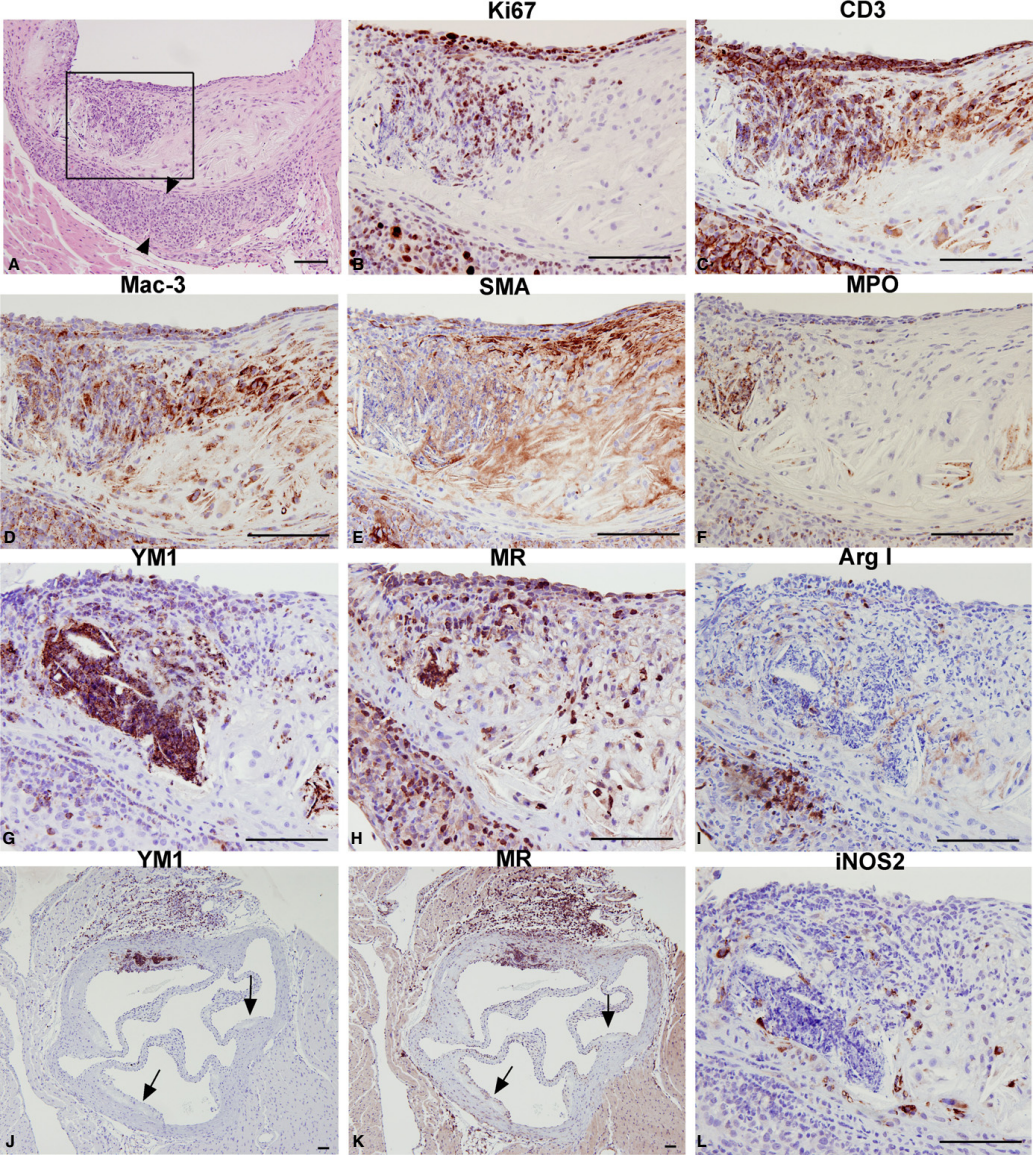
Cell‐type characterization of inflammatory cell infiltrates (ICIs). Low‐magnification hematoxylin and eosin staining delineates the intimal ICI in an intermediate lesion. (A, adventitial infiltrate is marked with arrowheads). Higher magnification consecutive sections of the ICI immunostained for Ki67 (B), T‐lymphocyte marker CD3 (C), macrophage marker Mac‐3, (D), smooth muscle cell actin (SMA) (E), neutrophil marker myeloperoxidase (MPO) (F), M2 macrophage marker chitinase 3‐like 3 (YM1) (G) mannose receptor (MR) (H), and arginase I (Arg I) (I) and M1 macrophage marker inducible nitric oxide synthase (iNOS) (L). YM1‐ and MR‐positive areas are limited to ICIs, both in the intima and adventitia, whereas lesions without the presence of acute inflammation do not express these markers (J and K, arrows). Bar=100 μm.

In addition to the identification of proliferating macrophages, prominent intimal ICIs containing proliferating cells (Figure 5A and 5B) were observed in some sections in the intermediate stage of lesion growth, typically associated with large apoptotic foci. These were not observed in early and advanced lesions. Large ICIs were also present in the adventitia underlying the intimal ICI (Figure 5A, arrowheads), and partially resemble previously described artery tertiary lymphoid organs (ATLOs).^17^, ^26^ Ki67 immunostaining showed that both the intimal and the adventitial ICIs were rich in proliferating cells (Figure 5B), most of which were CD3‐positive T lymphocytes (Figure 5C). They also contained macrophages (Mac‐3; Figure 5D), but very few smooth muscle cells (SMA; Figure 5E) or neutrophils (MPO; Figure 5F). Interestingly, the ICIs displayed high expression of the M2 markers, YM1 (Figure 5G) and MR (Figure 5H), while having low expression of the M2 marker, Arg 1 (Figure 5I), and the M1 marker, iNOS (Figure 5L). YM1‐ and MR‐positive cells were found solely in the ICIs, as indicated by lack of staining in other parts of the lesions (Figure 5J and 5K, arrows). Taken together, these findings suggest that T‐lymphocyte‐ and macrophage‐rich proliferating inflammatory infiltrates occur in the intima of intermediate lesions.

Subsequent to the formation of the necrotic core in advanced lesions, the presence of the ICIs diminished and correlated with few, if any, proliferating cells (Figure 1A and 1B, advanced). Eventually, the ICIs were completely resolved, both in the intima and in the adventitia of the advanced lesions, leaving behind a region containing dead tissue lacking nuclei and containing very few proliferating cells (Figure 1A, advanced). It has been reported that tertiary lymphoid follicles are found in human lesions and in the adventitia of old *Apoe* ^*−*/*−*^ mice.^26^, ^27^ To investigate these observations, we utilized IHC to determine whether ICIs represent an acute inflammatory response or lymphoid‐like follicles. In contrast to CD3‐positive T lymphocytes, the number of adventitial and intimal CD45R‐positive B cells was negligible, indicative of an acute inflammatory response in this region (Figure S3). Furthermore, as discussed above, the adventitial ICIs are transient in nature and do not exhibit the organized structure of lymphoid follicles. Our findings suggest that these novel lymphocytic aggregates or ICIs represent a transient innate immune reaction rather than organized ectopic lymphoid follicles.

To assess the presence of intimal ICIs during stages of lesion growth and their correlation with apoptosis, H&E‐stained sections from female *Apoe* ^*−/−*^ mice fed a chow diet (n=18) were visually examined for gross morphology. At 24 weeks of age, 5 of 18 mice showed the presence of ICIs both in the lesion and the underlying adventitia (Table). Only adventitial ICIs were present in an additional 5 mice. In contrast, 12‐week‐old female *Apoe* ^*−/−*^ mice fed a chow diet (n=15) demonstrated no signs of ICIs or apoptotic cell death. We also reviewed archival slides from a previous study characterizing a methionine model of accelerated lesion formation^20^ for the presence of ICIs and apoptosis. In 10‐week‐old female *Apoe* ^*−/−*^ mice fed a high methionine diet (n=15), which is known to accelerate lesion growth,^20^ 14 of 15 mice contained simple fatty streaks without apoptotic nuclei or ICIs. In 1 mouse, ICIs were observed around the apoptotic area and in the underlying adventitia. We believe that this represents the initial onset of apoptosis accompanied by an inflammatory response and formation of the intimal and adventitial ICIs. In addition, ICIs were assessed in 14‐week‐old male *LDLr* ^*−/−*^ mice fed a Western diet for 6 or 8 weeks (n=3 and 7, respectively). After 6 weeks on the diet, lesions were simple fatty streaks with no signs of apoptosis, necrosis, or ICIs. However, *LDLr* ^*−/−*^ mice fed a Western diet for 8 weeks presented with some lesions containing apoptotic bodies and cholesterol crystals. At this stage of lesion growth, ICIs were observed in the intima and underlying adventitia in 3 of the 7 mice studied (Figure S4, representative images). These data strongly support the notion that intimal ICIs are transient, exclusive to the intermediate stages of atherosclerosis, and correlate with apoptotic cell death.

**Table 1:** Quantification of ICIs in Mouse Models of Atherosclerosis

Strain	Age (weeks)	Diet	Intimal ICIs	Adventitial ICIs	Apoptosis
*Apoe* ^*−/−*^	24	Chow	5/18	10/18	Yes
*Apoe* ^*−/−*^	12	Chow	0/15	0/15	No
*Apoe* ^*−/−*^	10	Methionine	1/15	1/15	Yes (1)
*LDLr* ^*−/−*^	20	Western 6 weeks	0/3	0/3	No
*LDLr* ^*−/−*^	22	Western 8 weeks	3/7	3/7	Yes

Quantitative summary of several mouse groups that were scored for presence of intimal ICIs, adventitial ICIs, and apoptosis, showing a strong correlation between apoptosis and ICIs. Ratios depict number of animals exhibiting a certain parameter out of total mice analyzed. *Apoe* ^*−/−*^ animals were female and *LDLr* ^*−/−*^ animals were male. Five hematoxylin and eosin–stained sections along the aortic sinus, spaced 80 μm apart, were examined from each mouse (n=18 for 24‐week chow *Apoe* ^*−/−*^, n=15 for 12‐week chow *Apoe* ^*−/−*^, n=15 for 10‐week methionine *Apoe* ^*−/−*^, n=3 for 20‐week *LDLr* ^*−/−/−*^, and n=7 for 22‐week *LDLr* ^*−/−*^). ICIs indicates inflammatory cell infiltrates.

### Local Proliferation and Recruitment of T Lymphocytes in Inflammatory Cell Infiltrates

**Figure 6:**
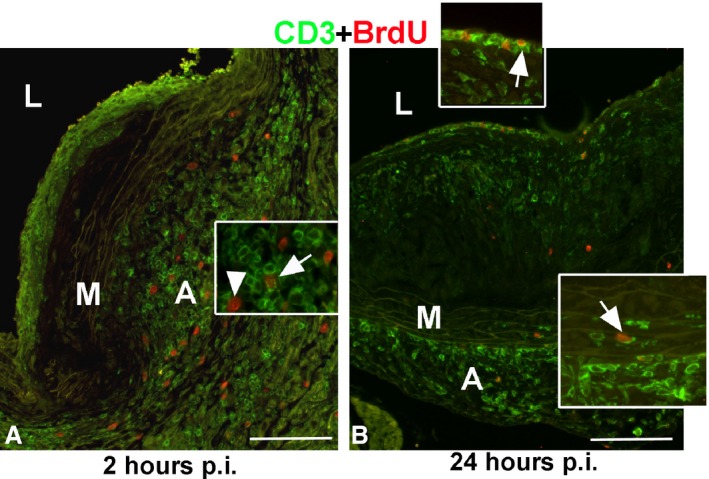
T‐cell recruitment into inflammatory cell infiltrates. Double immunofluorescence for CD3 (green) and BrdU (red). Two hours post‐BrdU injection, CD3‐positive lymphocytes in the intimal inflammatory cell infiltrate (ICI) were BrdU negative (A). BrdU‐positive nuclei were present in the adventitial ICI, some in T lymphocytes (arrow, A), and others in macrophages (arrowhead, A). At 24 hours p.i., some of the single‐file T lymphocytes lining the endothelium were BrdU positive (arrow, inset, B). Few CD3‐positive cells were observed in the media; 1 was BrdU positive (arrow, inset, B). L, lumen; M, media; A, adventitia. Bar=100 μm. BrdU indicates bromodeoxyuridine; p.i., postinjection.

BrdU pulse‐labeling and CD3 immunostaining was used to assess T‐lymphocyte recruitment into the ICIs. At 2 hours post‐BrdU pulse, CD3‐positive T lymphocytes in the intimal ICI lacked BrdU staining (Figure 6A), implying that the majority of these cells were derived from the circulation. Unlike the intima, BrdU‐positive T lymphocytes were observed in the underlying adventitial ICIs (Figure 6A, arrow). Other BrdU‐positive cells contained large nuclei and were negative for CD3 (Figure 6A, arrowhead). This suggests that T lymphocytes in intimal ICIs do not proliferate in situ, whereas some T lymphocytes in adventitial ICIs do. At 24 hours p.i., BrdU/CD3‐positive T lymphocytes were identified in the intima (Figure 6B, inset, arrow). The location of the BrdU/CD3‐positive cells that lie in a single‐file pattern along the intimal side of endothelium is suggestive of their recruitment from the aorta. A few BrdU/CD3‐positive cells were also observed in the media, suggesting that some of the proliferating T lymphocytes may cross the medial layer, most likely from the adventitia, into the internal part of the lesion (Figure 6B, lower inset, arrow). Overall, these data suggest that T lymphocytes proliferate locally in the adventitial space, whereas intimal ICI resident T lymphocytes accumulate from both the circulation and the adventitia before their active growth cycle.

### Inflammatory Cell Infiltrates Are Associated With Apoptosis and Resolve as Lesions Progress

**Figure 7:**
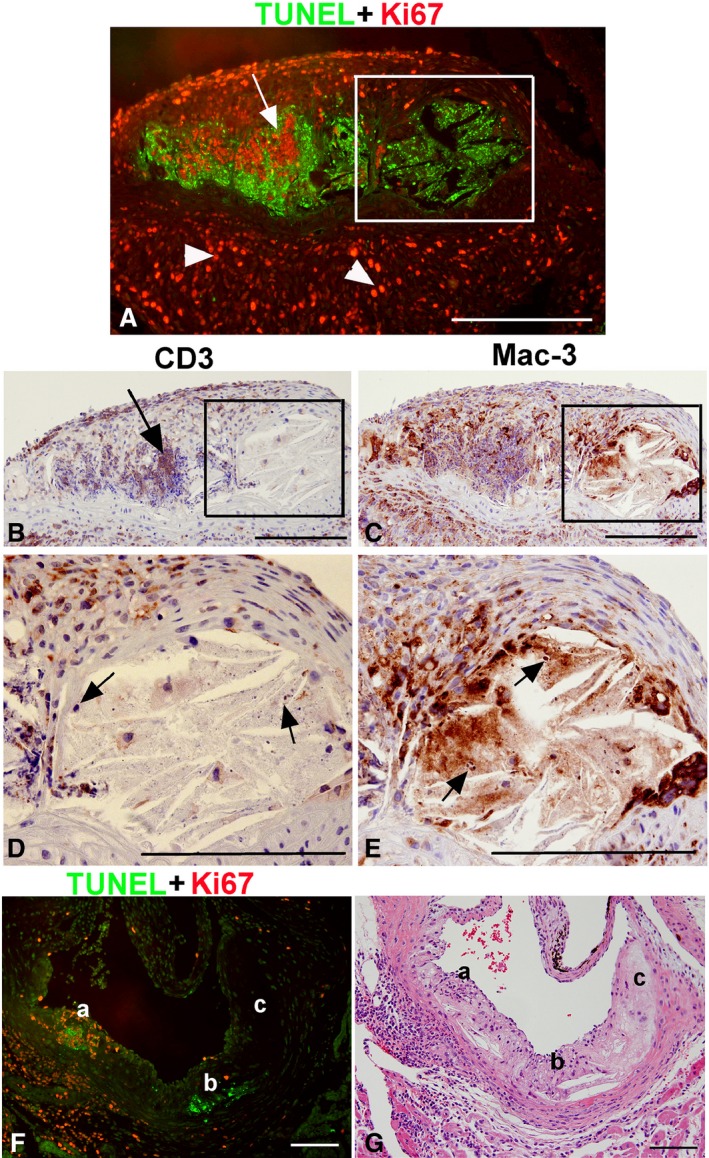
Association of inflammatory cell infiltrates with apoptosis. Double immunofluorescence for terminal deoxynucleotidyl transferase dUTP nick end labeling (TUNEL; green, A) and Ki67 (red, A). Consecutive sections immunostained for T lymphocytes (CD3, B and D) and macrophages (Mac‐3, C and E). Large numbers of cells in the adventitial inflammatory cell infiltrate (ICI) express Ki67 (A). The area of apoptosis is surrounded by proliferating cells, which are mostly T lymphocytes (arrows, A and B). In the adjacent necrotic area (inset), apoptotic cells and cellular debris with apoptotic bodies are clearly visible (D and E, arrows). These cells are identified as macrophages by Mac‐3 staining (E) and not T lymphocytes (D). In (F and G), a continuum of lesion progression from intermediate to necrotic core was observed and stained for apoptosis (TUNEL) and proliferation (Ki67), as well as hematoxylin and eosin. The earlier area of the lesion (a) is associated with ICIs in the intima and in the underlying adventitia and exhibits a lot of proliferation with some apoptosis. In a more‐advanced, but not quite necrotic, area of the lesion (b), ICIs are no longer present whereas TUNEL staining is positive. The very advanced atherosclerotic area (c) is composed of necrotic tissue with very few cells and lacks TUNEL staining and proliferative ICIs. Bars=100 μm.

As described previously, during the intermediate stage of lesion growth, intimal ICIs were typically found in regions containing apoptotic cell death, as measured by terminal deoxynucleotidyl transferase dUTP nick end labeling (TUNEL) staining (Figure 7A). These regions contained apoptotic bodies surrounded by CD3‐positive T lymphocytes (Figure 7B) and some macrophages (Figure 7C). In contrast to the intima, large adventitial ICIs contained CD3‐positive T lymphocytes with no apoptotic bodies (Figure 7A through 7C). In more‐advanced areas of the lesion, proliferation ceases (Figure 7A, box). This area containing apoptotic bodies and cellular debris was found to include macrophages (by Mac‐3 staining), but not T lymphocytes (Figure 7A, 7D, and 7E, arrows), suggesting that it is macrophages that undergo apoptosis, rather than T lymphocytes, in this region. This advanced area of the lesion progresses toward a necrotic core, as indicated by its acellular nature and lack of proliferation and ICIs. These data strongly suggest that resolution of ICIs precedes lesion advancement and necrotic core formation.

**Figure 8:**
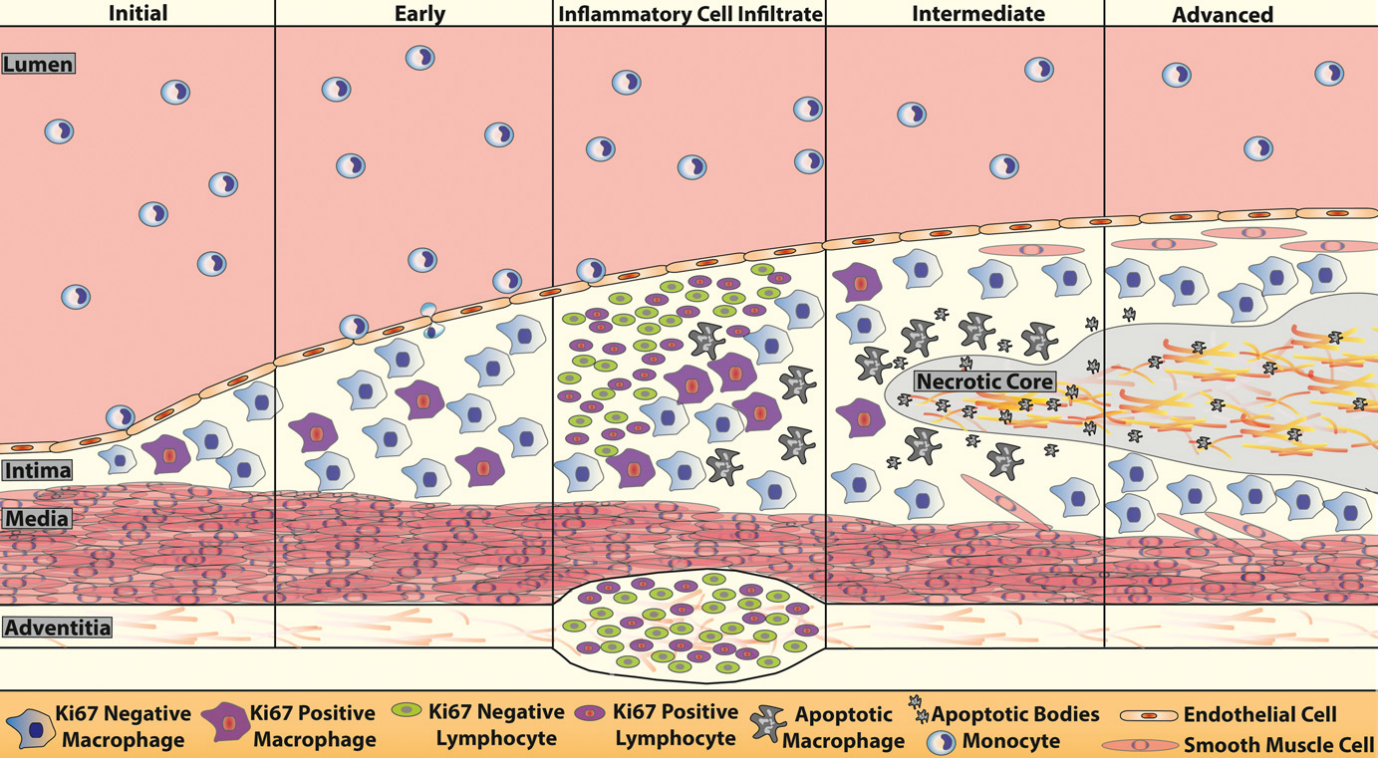
Summary diagram of macrophage proliferation and lymphocyte infiltration in a growing atherosclerotic lesion. Upon initiation of atherosclerosis, monocytes infiltrate the endothelial layer, differentiate into macrophages, and begin to proliferate and induce lesion growth. In early lesion formation, in situ macrophage proliferation increases and more monocytes are recruited. Lesions progress as lymphocyte involvement and immune activation become prevalent. Inflammatory cell infiltrates (ICIs) comprised of proliferating lymphocytes occur concurrently as macrophages undergo apoptosis as lesions transition from the early to the intermediate stage. The exact link behind the initiation of ICI formation and apoptosis is unknown, but could involve proinflammatory signaling. Adventitial ICIs or adventitial tertiary lymphoid organs are also observed and are a potential source for lymphocyte infiltration into the intima. As the intimal ICIs resolve, proliferation slows and lesions advance to the necrotic stage containg cellular debris and cholesterol crystals.

The different stages of ICI recruitment and necrotic core formation can also be detected in the intermediate stage of a representative complex lesion (Figure 7F and 7G). This diverse lesion includes the early apoptotic stage with proliferating ICIs (Figure 7F‐a), the later apoptotic stage where TUNEL‐positive apoptotic bodies are present but the acute ICIs have been resolved (Figure 7F‐b), and, finally, a necrotic core with the absence of cells/nuclei (Figure 7F‐c). These stages of ICI recruitment and necrotic core formation are clearly observed in a consecutive H&E section (Figure 7G). Taken together, these observations suggest that the intimal ICIs may be induced during early apoptosis in the lesion by recruiting inflammatory cells from the aorta and/or the adventitia. This advancing lesion continuum of macrophage proliferation, apoptosis, ICI formation, and resolution and subsequent necrosis is depicted as a model in Figure 8.

## Discussion

In this report, we demonstrate that in situ macrophage proliferation is prevalent in both murine and human atherosclerosis, with the greatest amount of locally proliferating macrophages occurring in early lesions. Notably, we identify the presence of intimal ICIs enriched with proliferating T lymphocytes in actively growing lesions and show their correlation with apoptosis.

The classical notion that macrophages in lesions are exclusively recruited from circulating monocytes, differentiate terminally, and stop dividing has recently been challenged and addressed extensively.^7^, ^12^ Interestingly, mitotic activity was reported in foam cells as early as 1948, and despite this process being continuously investigated, only recently has the focus shifted toward fully understanding the proliferative and apoptotic dynamics of macrophages within lesions.^4^, ^10^, ^28^ By using BrdU and Ki67 staining, the recent study by Robbins et al. demonstrated that macrophage self‐renewal contributes significantly to the macrophage population within the lesion.^9^ Based on these findings, our study was designed to systematically characterize local macrophage proliferation at various stages of lesion development and to identify other lesion‐resident proliferating cells and their correlation with apoptosis and plaque progression.

Ki67 has been extensively utilized to demonstrate lesion resident macrophage proliferation at various stages of human atherosclerosis, and it enables for accurate detection of cells that are actively proliferating that are not in the G_0_ phase of the cell cycle.^22^, ^29^, ^30^ We identified proliferating cells at various stages of lesion development in *Apoe* ^*−/−*^ mice fed a chow diet, with the predominant amount of cells appearing in the early (12 weeks old) and intermediate (24 weeks old) stages of atherosclerosis. We utilized the *Apoe* ^*−/−*^ animal model on a chow diet because of its relevance to human disease development and its gradual nature of plaque maturation, enabling us to accurately assess cellular proliferation at distinct stages of lesion progression.^31^, ^32^ Furthermore, we showed the presence of Ki67 proliferating cells in human coronary artery lesions and demonstrated that these proliferating cells are primarily macrophages in both human and murine plaques. This highlights the prevalence of proliferating cells in atherosclerosis and may represent a universal phenomenon among different species. These findings are consistent with those of Robbins et al. and give credence to investigating and characterizing in situ proliferation in our models.^9^

Although Ki67 is a useful, well‐characterized marker of cell proliferation, its use in identifying the source of proliferating cells is limited.^22^ Alternatively, utilizing a pulse labeling of BrdU allows for accurate identification of the origin of proliferating cells.^24^ By comparing BrdU‐positive cells at 2 and 24 hours p.i. in aortic sections, we were able to estimate the relative contribution of in situ proliferation and monocyte recruitment into the lesion. In short, any cells that are positive at 2 hours p.i. are considered to have been labeled in situ and are not derived from circulating monocytes. Robbins et al. utilized osmotic pumps that continually saturated cells with BrdU, thus complicating the source of the positively stained proliferating cells, especially given that BrdU is maintained in daughter cells.^1^, ^9^ This method thus allowed us to quantitatively assess the exact contribution of in situ proliferating macrophages and those derived from the circulation and determine that 43% of mitotic macrophages are derived from local proliferation in early lesions. Furthermore, using our BrdU method, we showed, both visually and quantitatively, that overall proliferation decreases as lesions advance from an early to intermediate state. This notion differs from other views of in situ macrophage proliferation, which state that local proliferation is predominant at later stages whereas monocyte recruitment is prevalent in early murine atherosclerosis.^9^, ^10^ Interestingly, Lutgens et al. showed that in humans, DNA synthesis is primarily an early lesion event that slowly halts and gets replaced by apoptosis as lesions advance.^30^ Our BrdU pulse‐labeling proliferation data supports this concept and reinforces our use of *Apoe* ^*−/−*^ mice on a chow diet in analyzing the dynamics of macrophage proliferation, as opposed to other, more‐accelerated diet‐induced models of atherosclerosis. It is evident that local macrophage proliferation is a prevalent phenomenon in early atherosclerotic lesions and studying this dynamic cellular biology in more detail would significantly enhance understanding of the disease process as a whole. We therefore also illustrate a technique using Ki67 and BrdU co‐immunostaining to accurately identify cell‐cycle stages of proliferating macrophages (Figure S5 and Data S1), which can be used to study this process at a greater depth.

Interestingly, we also observed several cells in various lesions that were positive for Ki67, but were not macrophages, a finding that we further investigated. One striking observation was the presence of intimal ICIs in intermediate lesions that contained large cores of proliferative cells. This diverse milieu of proliferating cells represents the first report of cellular proliferation in *intimal* inflammatory infiltrates of atherosclerotic lesions. To our knowledge, inflammatory infiltrates are usually described as adventitial phenomena, often containing B cells and termed ATLOs.^26^, ^33^ They have seldom been observed in the intima, with only 1 such report acknowledging them in arteritis of renal allograph patients.^34^ We determined that these ICIs were comprised primarily of proliferating T lymphocytes and lacked B cells. Proliferation of T lymphocytes has been described in the adventitia with a report addressing the influence of antigen presenting cells and cytokines on this cell subset.^27^, ^35^ Moreover, though the migration and proinflammatory effects of T lymphocytes in the arterial wall has been explored,^17^ no study has addressed proliferation of T lymphocytes in the intima or examined defined stages of lesion formation. Our findings visually illustrate the proliferation of intimal T lymphocytes in ICIs and led us to explore the potential functional implications of this unique, albeit transient, phenomenon.

These ICIs were only identified in our gradual onset models of atherosclerosis progression and disappeared in mature lesions, thus pointing to their occurrence being short‐lived. In fact, our ability to separate the different stages of atherosclerosis in *Apoe* ^*−/−*^ mice fed a chow diet allowed us to identify these ICIs, which comprise an important stage of lesion progression. Furthermore, this also enabled the identification of apoptosis adjacent to the ICIs and the observation that ICIs disappeared as lesions advanced and necrotic cores formed. Interestingly, ICIs and apoptotic foci were absent in early lesions and only occurred sporadically in intermediate lesions. This suggests that the acute inflammatory stage containing ICIs is likely to be transient in nature and contains an acute phase of recovery. Nevertheless, we show that T‐lymphocyte‐enriched ICIs correlate with apoptosis and precede necrosis in lesions upon resolution (Figure 8).

We also sought to characterize the T lymphocytes contained in the ICIs as either cytotoxic/proinflammatory (CD8 positive) or as helper/regulatory cells (CD4 positive).^36^, ^37^ We identified that the majority of T lymphocytes in intimal ICIs were CD8 and CD4 negative while being CD3 positive (Figure S6), which represent a novel class of T lymphocytes termed double‐negative T (DNT) cells.^38^ DNT cells have been implicated in autoimmune disorders and transplantation immunology with potential roles in apoptosis.^38^, ^39^ The molecular connection between DNT cells and lesion apoptosis is not known; however, to our knowledge, we are the first to identify DNT cells in atherosclerotic lesions and show they comprise the majority of the ICI.

Overall, our findings strongly suggest that ICIs are induced during the actively growing phase of lesion development and contribute to the process of localized apoptosis within the intimal space, potentially through recruitment of inflammatory cells. As lesions advance, apoptotic cells cannot be cleared as macrophage foam cells lose their ability to phagocytose, thereby further propagating inflammation.^6^ Necrosis subsequently occurs and results in the formation of necrotic cores,^40^ with lesion‐resident ICIs disappearing before this stage. Figure 7F is a unique example of 3 stages of lesion growth and necrotic core formation occurring simultaneously in 1 section; a—the early apoptotic stage with ICI containing proliferating Ki67‐positive cells in the lesion and in the underlying adventitia; b—late apoptotic stage where apoptotic bodies are present but acute inflammation has been resolved; and c—necrotic core with absence of cells/nuclei. This delineates how proliferating T lymphocytes comprising the ICI, and particularly DNT cells, can influence apoptosis and foam cell dynamics as lesions progress. Furthermore, ICIs represent an acute inflammatory event that is rapid and short‐lived and may often be missed by histochemical methods. These previously uncharacterized proliferating and biologically active ICIs comprise an important and relevant stage of atherosclerotic growth that may contribute to apoptotic mechanisms and necrosis (Figure 8).

Proliferating atherosclerotic lesion‐resident cells remain largely uncharacterized and poorly understood despite their importance in the progression of CVD. We have shown the prevalence and significance of in situ proliferating macrophages at various stages of murine and human atherosclerotic lesions using Ki67 staining and BrdU pulse‐labeling experiments across several models of atherosclerosis. Furthermore, we have identified T‐lymphocyte‐enriched ICIs as a novel transient inflammatory event during active lesion growth. These findings will give rise to a better understanding of human atherosclerosis at the cellular level, particularly with regard to macrophage dynamics, in situ proliferation of leukocytes, as well as apoptosis and necrosis. Overall, this work establishes macrophage and T‐lymphocyte self‐renewal as a major contributor to lesion growth and stability and as a potential novel therapeutic target for atherosclerosis.

## Sources of Funding

This work was supported, in part, by research grants to Richard C. Austin from the Heart and Stroke Foundation of Canada (G‐15‐0009389), the Canadian Institutes of Health Research (MOP‐286787), and a Heart and Stroke Foundation of Ontario Program Grant (PRG6502). Financial support from St. Joseph's Healthcare Hamilton is acknowledged. Richard C. Austin is a Career Investigator of the Heart and Stroke Foundation of Ontario and holds the Amgen Canada Research Chair in the Division of Nephrology at St. Joseph's Healthcare and McMaster University.

## Disclosures

None.

## Supporting information


**Data S1.** Utilization of Ki67 and BrdU co‐immunostaining to identify stages of proliferation of lesion macrophages.
**Figure S1.** Lipid content in *Apoe* ^*−/−*^ lesions. Phase contrast (A), filipin staining for free cholesterol (B) and Oil Red O (ORO) for neutral lipids (C) in early lesion. Higher magnification of early lesion filipin staining (D) and corresponding ORO (E) in an early lesion, and higher magnification of advanced lesion filipin (F) and ORO (G).
**Figure S2.** BrdU immunohistochemistry in small intestine from mice injected with BrdU. Paraffin sections of the jejunum were isolated from *Apoe* ^*−/−*^ mice stained with BrdU. At 2 hours p.i., cells in the crypts were positive, whereas at 24 hours after the BrdU pulse, labeled cells have migrated up the villus. BrdU indicates bromodeoxyuridine; p.i., postinjection.
**Figure S3.** B‐cell immunofluorescence in intimal and adventitial inflammatory cell infiltrates. Double immunofluorescence for T lymphocytes (CD3, red) and B cells (CD45R, green) on paraffin sections of intermediate lesions from *Apoe* ^*−/−*^ mice showed minor staining for B cells and abundance of CD3‐positive T lymphocytes. Double fluorescence for Ki67 (red) and CD45R showed that B cells do not proliferate in these regions.
**Figure S4.** Intimal and adventitial inflammatory cell infiltrates (ICIs) in *LDLr* ^*−/−*^ mice. Hematoxylin and eosin staining of lesions from *LDLr* ^*−/−*^ mice on high‐fat diet for 8 weeks show the presence of both intimal and adventitial ICIs (arrows).
**Figure S5.** Comparison of lesion‐resident BrdU‐labeled and Ki67‐immunopositive macrophages. BrdU marks the cell in S phase during the pulse, whereas Ki67 positivity represents expression of the protein at the time of sacrifice. At 2 hours p.i., cells are either double positive (A, arrow) or Ki67 positive only (A, arrowhead). At 24 hours p.i., in addition to the double‐positive cells (B, arrow) and Ki67 positive only (B, arrowhead), some cells are BrdU positive only (B, double arrow). These represent daughter cells that were in the G_0_ phase at the time of sacrifice. Bar=50 μm. BrdU indicates bromodeoxyuridine; p.i., postinjection.
**Figure S6.** T lymphocytes in inflammatory cell infiltrates (ICIs) are CD3^+^CD4^−^CD8^−^ (double‐negative T cells). Double immunofluorescence for CD8 (green) and CD3 (red) in lesions with ICIs in *Apoe* ^*−/−*^ mice (A) and in the thymus as a positive control (B). Only a few of the CD3^+^ cells in the ICIs were CD8^+^ (inset in A, arrow). Immunohistochemistry for CD4 in ICI in *Apoe* ^*−/−*^ lesion (C) and in the thymus as a positive control (D). Very few of the T lymphocytes in the lesion (arrows, inset) and in the underlying adventitia (arrowheads) were CD4 positive. L, lumen. Bar=100 μm (A and B); Bar=50 μm (C and D).
