# Supplementary material for: Characterization of Proliferating Lesion‐Resident Cells During All Stages of Atherosclerotic Growth
Source: J Am Heart Assoc. 2016 Aug 15;5(8):e003945. doi: 10.1161/JAHA.116.003945 (PMC5015311; doi:10.1161/JAHA.116.003945)
Supplement: Supplementary file 1 — Data S1. Utilization of Ki67 and BrdU co‐immunostaining to identify stages of proliferation of lesion macrophages. Figure S1. Lipid content in Apoe −/− lesions. Phase contrast (A), filipin staining for free cholesterol (B) and Oil Red O (ORO) for neutral lipids (C) in early lesion. Higher magnification of early lesion filipin staining (D) and corresponding ORO (E) in an early lesion, and higher magnification of advanced lesion filipin (F) and ORO (G). Figure S2. BrdU immunohistochemistry in small intestine from mice injected with BrdU. Paraffin sections of the jejunum were isolated from Apoe −/− mice stained with BrdU. At 2 hours p.i., cells in the crypts were positive, whereas at 24 hours after the BrdU pulse, labeled cells have migrated up the villus. BrdU indicates bromodeoxyuridine; p.i., postinjection. Figure S3. B‐cell immunofluorescence in intimal and adventitial inflammatory cell infiltrates. Double immunofluorescence for T lymphocytes (CD3, red) and B cells (CD45R, green) on paraffin sections of intermediate lesions from Apoe −/− mice showed minor staining for B cells and abundance of CD3‐positive T lymphocytes. Double fluorescence for Ki67 (red) and CD45R showed that B cells do not proliferate in these regions. Figure S4. Intimal and adventitial inflammatory cell infiltrates (ICIs) in LDLr −/− mice. Hematoxylin and eosin staining of lesions from LDLr −/− mice on high‐fat diet for 8 weeks show the presence of both intimal and adventitial ICIs (arrows). Figure S5. Comparison of lesion‐resident BrdU‐labeled and Ki67‐immunopositive macrophages. BrdU marks the cell in S phase during the pulse, whereas Ki67 positivity represents expression of the protein at the time of sacrifice. At 2 hours p.i., cells are either double positive (A, arrow) or Ki67 positive only (A, arrowhead). At 24 hours p.i., in addition to the double‐positive cells (B, arrow) and Ki67 positive only (B, arrowhead), some cells are BrdU positive only (B, double arrow). These represent daughter c [file JAH3-5-e003945-s001.pdf]

SUPPLEMENTAL MATERIAL

Characterization of Proliferating Lesion-Resident Cells during All Stages of Atherosclerotic Growth

Running title: *Proliferating cells in atherosclerotic lesions*

Šárka Lhoták, PhD[1,6]; Gabriel Gyulay, PhD[1,6], Jean-Claude Cutz, MD[2]; Ali Al-Hashimi PhD[1,6]; Bernardo L. Trigatti, PhD[3]; Carl D. Richards, PhD[2]; Suleiman A. Igdoura, PhD[4]; Gregory R. Steinberg, PhD[5]; Jonathan Bramson, PhD[2]; Kjetil Ask, PhD[3]; Richard C. Austin, PhD[1,6]

[1]Department of Medicine, Division of Nephrology, McMaster University, Hamilton, Ontario, Canada; [2]Department of Pathology and Molecular Medicine, McMaster University, Hamilton, Ontario, Canada; [3]Department of Biochemistry and Biomedical Sciences, McMaster University, Hamilton, Ontario, Canada; [4]Department of Biology, McMaster University, Hamilton, Ontario, Canada; [5]Department of Medicine, Division of Endocrinology, McMaster University, Hamilton, Ontario, Canada; [6]Hamilton Centre for Kidney Research, St. Joseph's Healthcare Hamilton, Hamilton, Ontario, Canada

Data S1

Utilization of Ki67 and BrdU co-immunostaining to identify stages of proliferation of lesion macrophages

Ki67 is expressed in cells during all stages of cell cycle, from G_1 phase through to G_2/M, but not in quiescent cells (G_0) [1]. Thus, Ki67 immunopositivity represents lesion-resident cells expressing Ki67 protein at the time of tissue fixation. In contrast, BrdU is incorporated into newly synthesized DNA (S phase) during the pulse phase and as a result, Ki67 staining identifies more lesion-resident proliferating macrophages, compared to BrdU incorporation. Double immunofluorescence revealed BrdU/Ki67 positive macrophage foam cells 2 hours post-injection (Figure S5A, arrow). However, cells that were not in S phase during the pulse but in the G1 or G2/M phase during fixation, were only positive for Ki67, as expected (Figure S5A, arrowhead). At 24 hours post-injection, in addition to the BrdU/Ki67 positive cells (Figure S5, arrow) and Ki67-only positive cells (Figure S5B, arrowhead), some BrdU-only positive cells were observed (Figure 5B, double arrowhead). These represent daughter cells that were in S phase 24 hours before sacrifice, had completed their division and were in G_0 phase with undetectable Ki67 expression at the time of sacrifice, indicative of cells that completed the cell cycle within 24 hours. These findings show that the combination of Ki67 and BrdU incorporation can be used to identify proliferating macrophages at different stages of the cell cycle.

SUPPLEMENTAL FIGURES

Figure S1

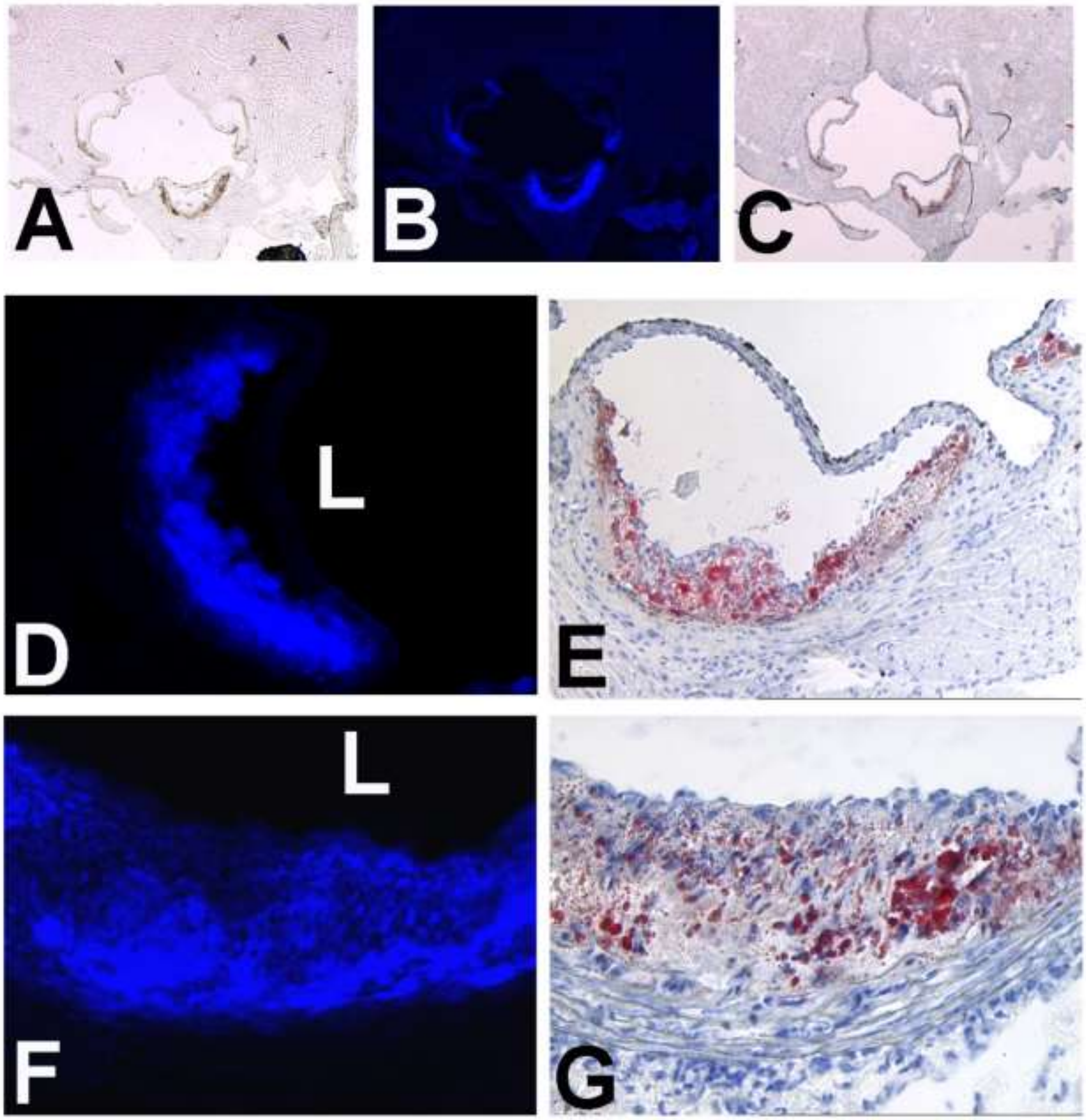

Figure S2

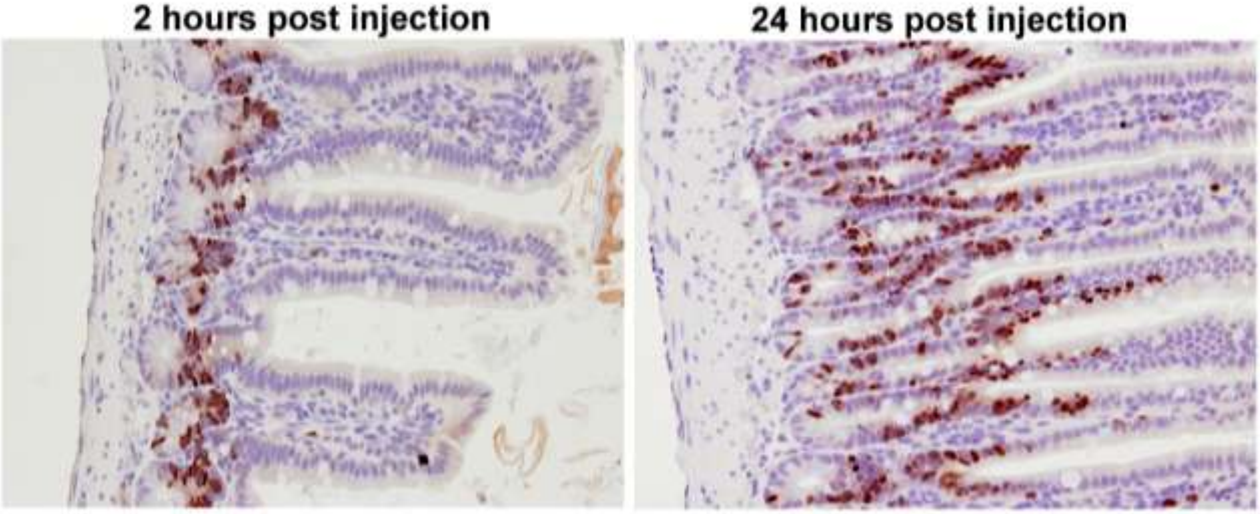

Figure S3

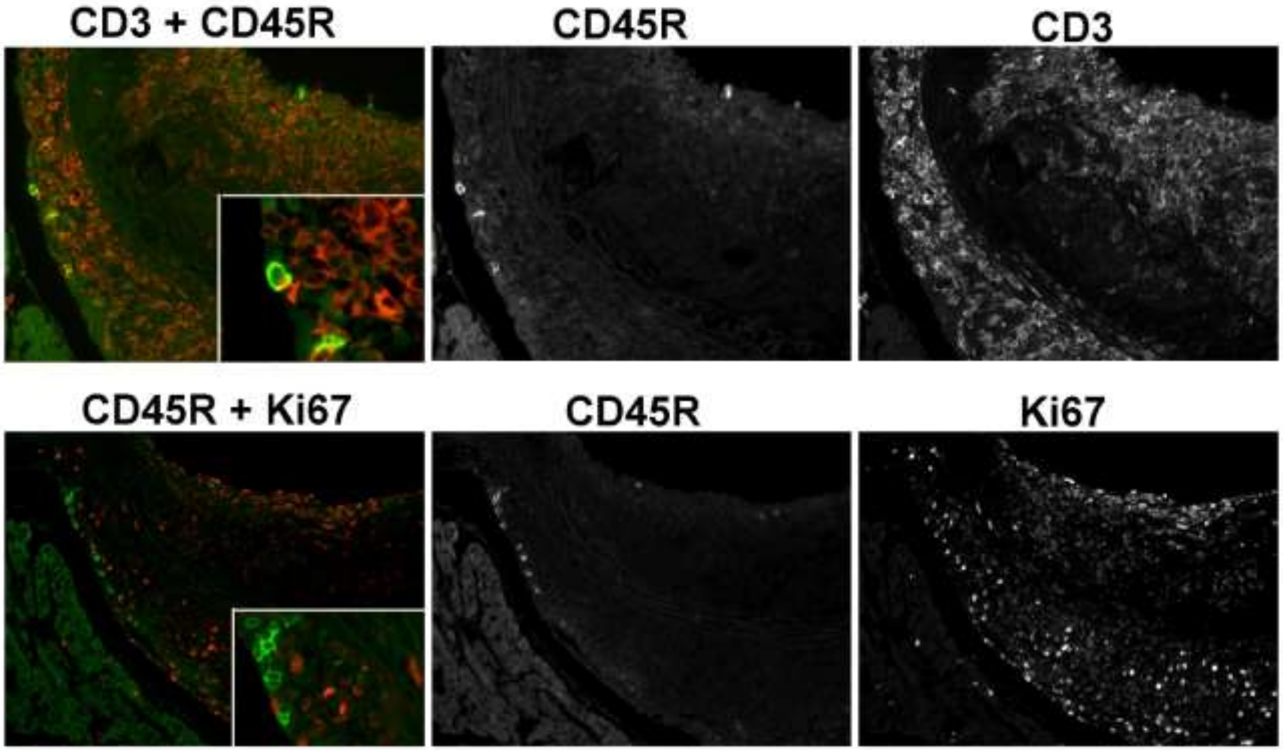

Figure S4

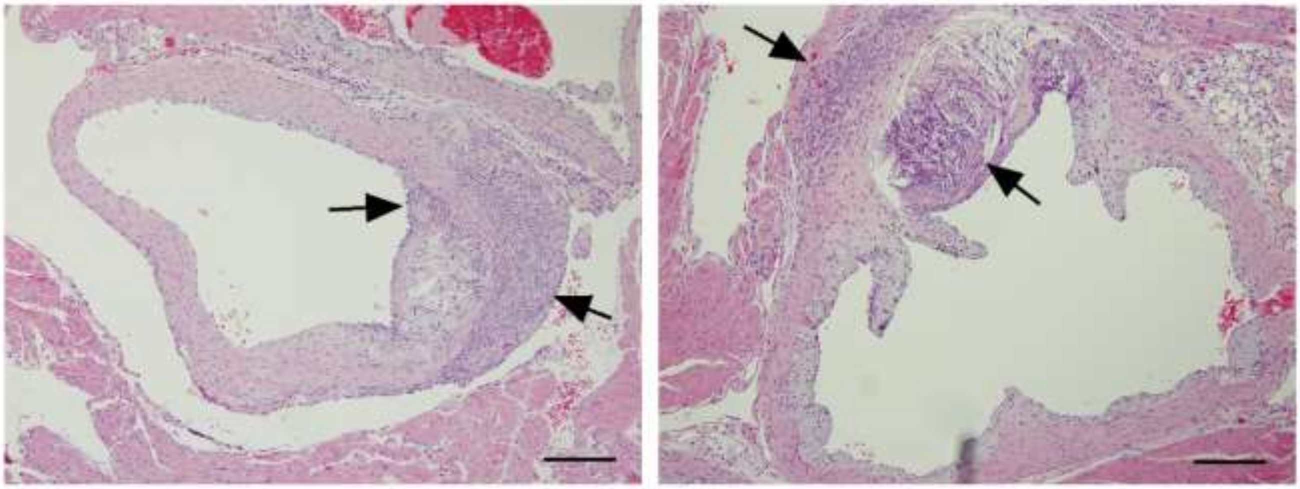

Figure S5

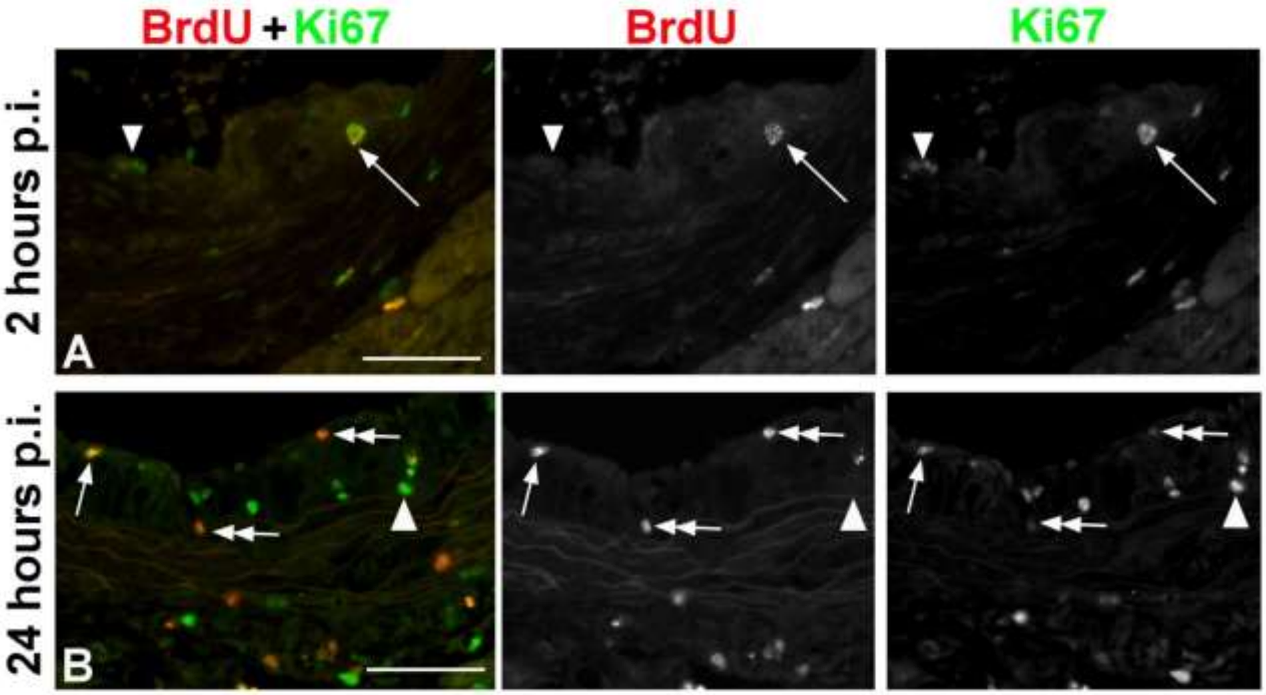

Figure S6

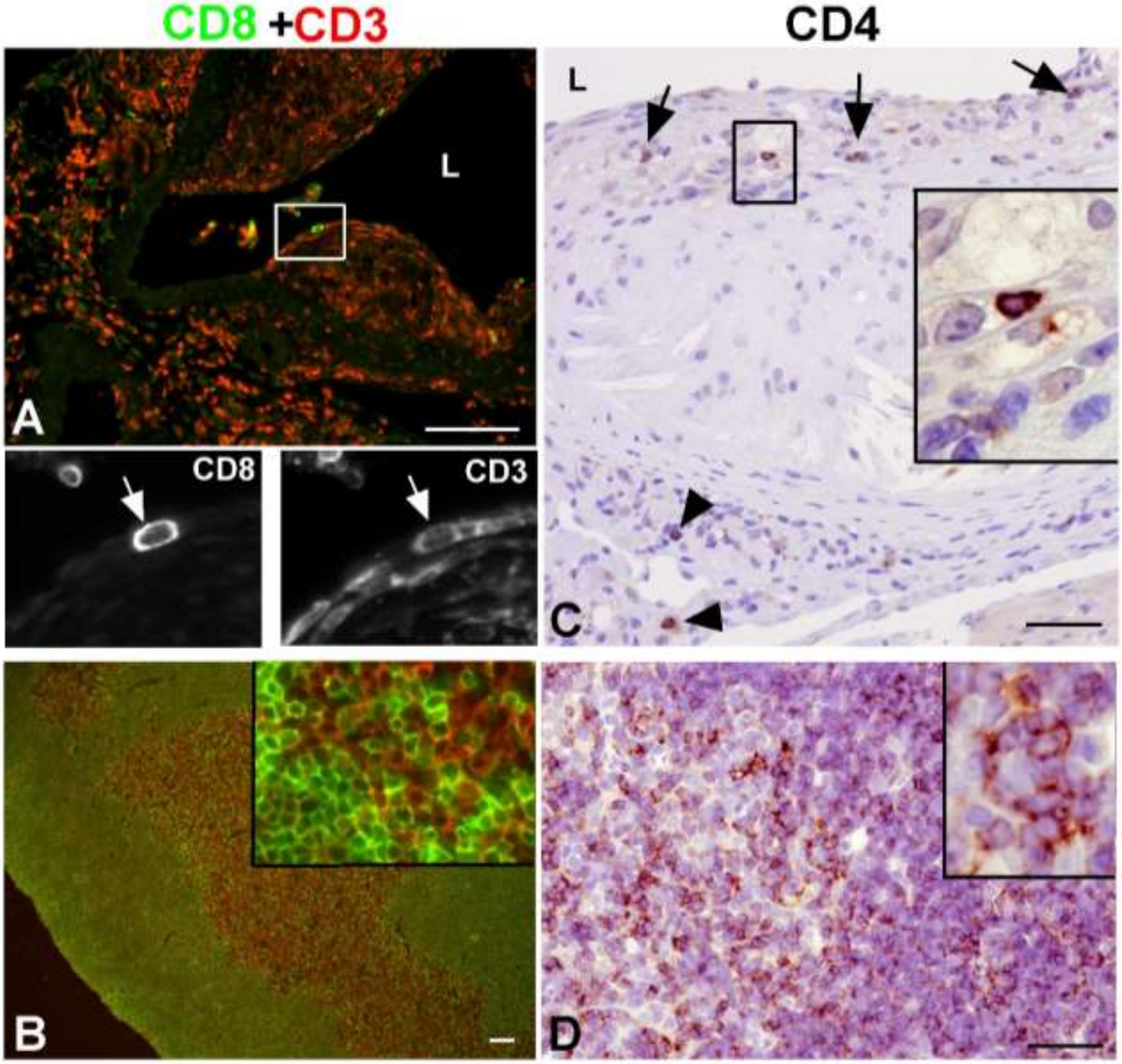

SUPPLEMENTAL FIGURE LEGENDS

Figure S1. Lipid content in *Apoe*$^{-/-}$ lesions.

Phase contrast (**A**), filipin staining for free cholesterol (**B**) and Oil Red O for neutral lipids (**C**) in early lesion. Higher magnification of early lesion filipin staining (**D**) and corresponding oil red O (**E**) in an early lesion, and higher magnification of advanced lesion filipin (**F**) and ORO (**G**).

Figure S2. BrdU immunohistochemistry in small intestine from mice injected with BrdU. Paraffin sections of the jejunum were isolated from *Apoe*$^{-/-}$ mice stained with BrdU. At 2 hours p.i., cells in the crypts were positive, whereas at 24 hours after the BrdU pulse, the labeled cells have migrated up the villus.

Figure S3. B cell immunofluorescence in intimal and adventitial inflammatory cell infiltrates.

Double immunofluorescence for T-lymphocytes (CD3, red) and B cells (CD45R, green) on paraffin sections of intermediate lesions from *Apoe*$^{-/-}$ mice showed minor staining for B cells and abundance of CD3 positive T-lymphocytes. Double fluorescence for Ki67 (red) and CD45R showed that B cells do not proliferate in these regions.

Figure S4. Intimal and adventitial ICIs in *LDLr*$^{-/-}$ mice.

H&E staining of lesions from *LDLr*$^{-/-}$ mice on high fat diet for 8 weeks show the presence of both intimal and adventitial ICIs (arrows).

Figure S5. Comparison of lesion-resident BrdU labeled and Ki67 immunopositive macrophages.

BrdU marks the cell in S phase during the pulse, whereas Ki67 positivity represents the expression of the protein at the time of sacrifice. At 2 hours p.i. cells are either double positive (**A**, arrow) or Ki67 positive only (**A**, arrowhead). At 24 hours p.i., in addition to the double positive cells (**B**, arrow) and Ki67 positive only (**B**, arrowhead), some cells are BrdU positive only (**B**, double arrow). These represent daughter cells that were in the G0 phase at the time of sacrifice. Bar=50 µm.

Figure S6. T-lymphocytes in ICIs are $CD3^{+}CD4^{-}CD8^{-}$ (Double Negative T-cells). Double immunofluorescence for CD8 (green) and CD3 (red) in lesions with ICIs in *Apoe*$^{-/-}$ mice (**A**), and in the thymus as a positive control (**B**). Only a few of the $CD3^{+}$ cells in the ICIs were $CD8^{+}$ (inset in **A**, arrow). IHC for CD4 in ICI in *Apoe*$^{-/-}$ lesion (**C**) and in the thymus as a positive control (**D**). Very few of the T-lymphocytes in the lesion (arrows, inset) and in the underlying adventitia (arrowheads) were CD4 positive. L; lumen. Bar=100 µm (**A,B**); Bar=50 µm (**C, D**).

REFERENCES

1. Scholzen T and Gerdes J. The Ki-67 protein: from the known and the unknown. *Journal of cellular physiology*. 2000;182:311-22.
